# Prof. Dan’l F. Wright, M. D.

**Published:** 1858-08

**Authors:** 


### Prof. Dan'l F. Wright, M. D.

Prof. Dan'l F. Wright, M. D., the recent distinguished occupant of the Chair of Physiology, and Pathology in the Memphis Medical College, has resigned that position, and accepted the same Chair in the Shelby Medical College, at Nashville, Tennessee.

